# Images in infectious diseases: generalized Herpes simplex—images of an unusual HIV first presentation

**DOI:** 10.1007/s15010-023-02065-0

**Published:** 2023-07-05

**Authors:** Benjamin T. Schleenvoigt, Anne Moeser, Dragiša Mitić, Michael Baier, Christoph Stephan, Anna Pothmann

**Affiliations:** 1 Institute of Infectious Diseases and Infection Control, Jena University Hospital/Friedrich-Schiller-University, Jena, Germany; 2 Institute for Medical Microbiology, Jena University Hospital/Friedrich-Schiller-University, Jena, Germany; 3 Center of Internal Medicine, Infectious Diseases Unit, University Hospital Frankfurt, Goethe University, Frankfurt, Germany; 4 Department of Dermatology, Jena University Hospital/Friedrich-Schiller-University, Jena, Germany

A 43-year-old Serbian heterosexual man, who migrated to Germany 2 months before, presented in February 2023 because of HIV initial diagnosis. HIV viral load and CD4 count were 787,110 copies/ml and 2 cells/µl, respectively. On clinical examination, a pruritic disseminated rash consisting of excoriated erythematous nodules, papules and vesicules was evident (Image 1A–G), skin PCR swaps tested positive for Herpes simplex virus-1 (HSV-1)—without findings for HSV-2, varicella zoster virus (VZV) and Monkeypox. Results for Herpes virus PCR from blood (HSV-1, HSV-2 and VZV) were negative. Measels (IgG) and Lues-serology (TPPA, VDRL) were unremarkable in terms of previous infections.

**Image 1:**
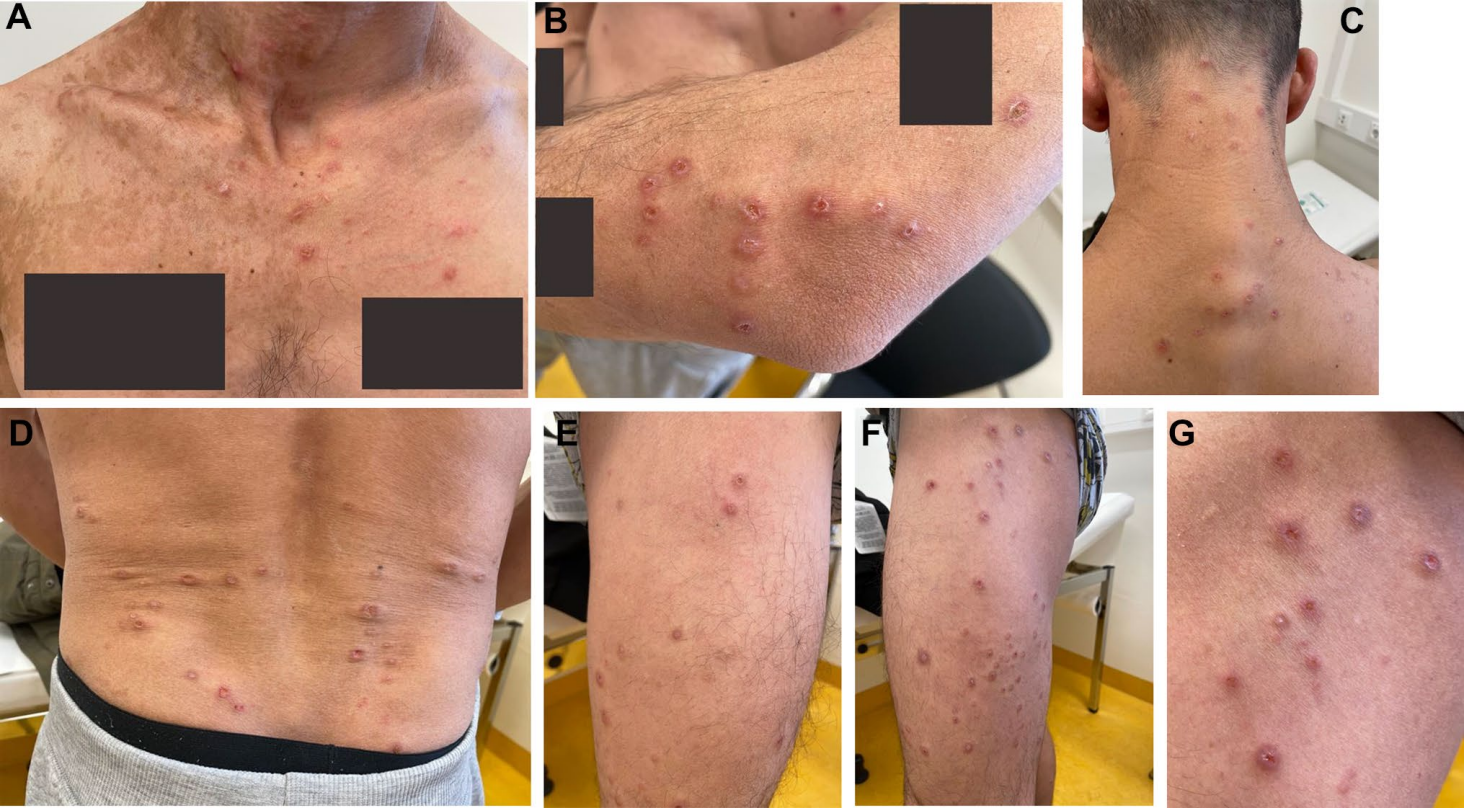
**A**–**G** Generalized herpes simplex of the skin on first presentation of HIV-Infection in an 43-year-old male from Serbia

HIV-infection was treated with Bictegravir/Tenofovir–Alafenamid/Emtricitabin (50/25/200 mg) per os qd and PcP-prophylaxis with Cotrimoxazol 480 mg per os qd was initiated. Since no other opportunistic infections were found, no clinical neurological deficit was detectable and a systemic HSV-1 infection was ruled out, the patient was treated as an outpatient with Aciclovir 400 mg 5 × daily per os  [1]. The patient applied Betamethasone/fusidic acid ointment on affected skin lesions. After 3 days, antiviral treatment was switched to Valaciclovir 1000 mg tid for another 7 days, due to pharmacokinetic considerations [2]. During follow-up—16 days after initial presentation, the patient showed signs of insufficient drug adherence, due to insufficient understanding of the mode action, in the context of illiteracy. Moreover, no satisfying improvements in his skin condition could be observed (Image 2A–I). With the aid of a Serbian native speaker, the patient was trained to better adhere to his regimen, and a second course of Valaciclovir for 7 days was prescribed. Re-examination after 13 days (29 days after initial presentation) revealed good adherence. HIV viral load decreased to 4.700 copies/ml, and CD4-count raised to 29 cells/µl, respectively. The HSV-1-related lesions finally healed after two courses of valaciclovir with scaring (Image 3A–F).

**Image 2:**
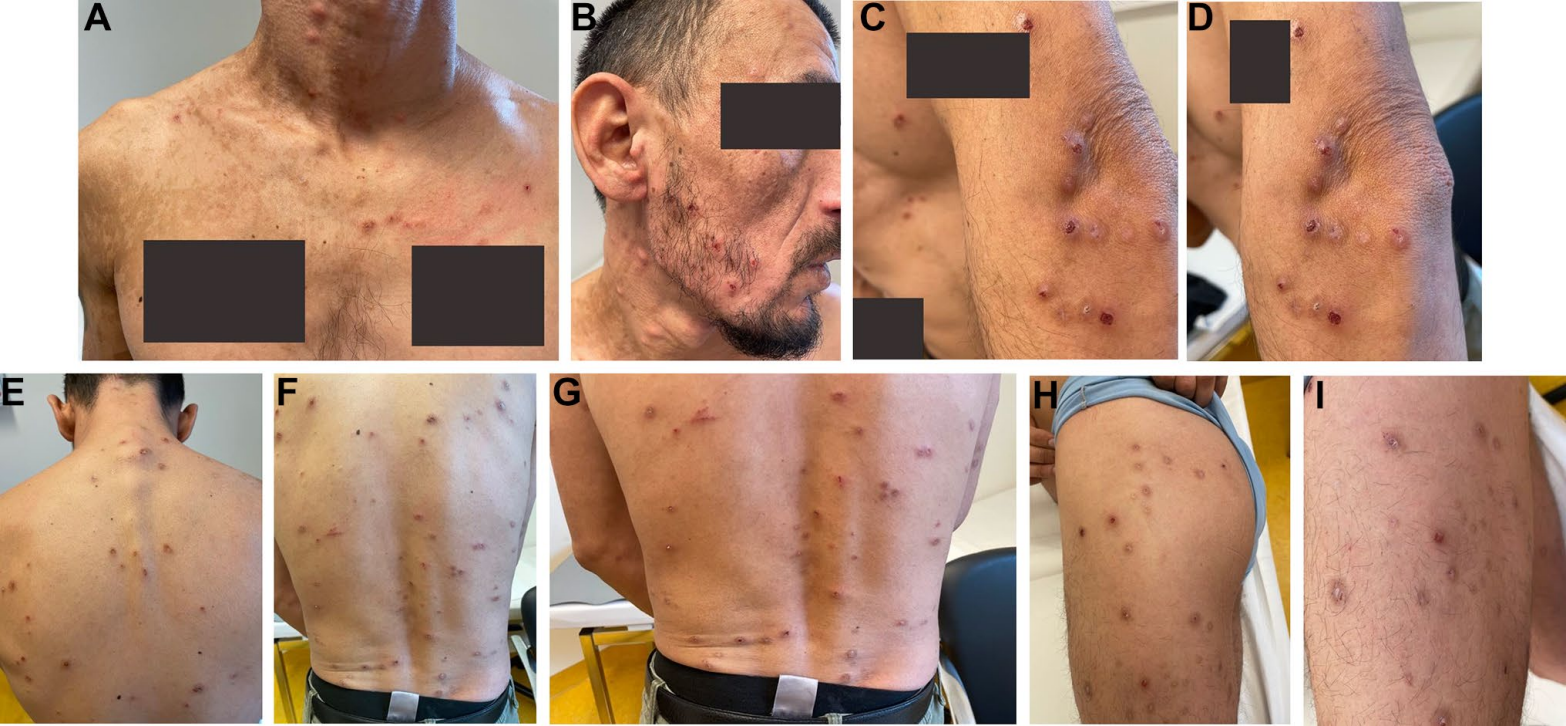
**A**–**I** Clinical course after first course of treatment with Valaciclovir with compliance problems due to illiteracy

**Image 3:**
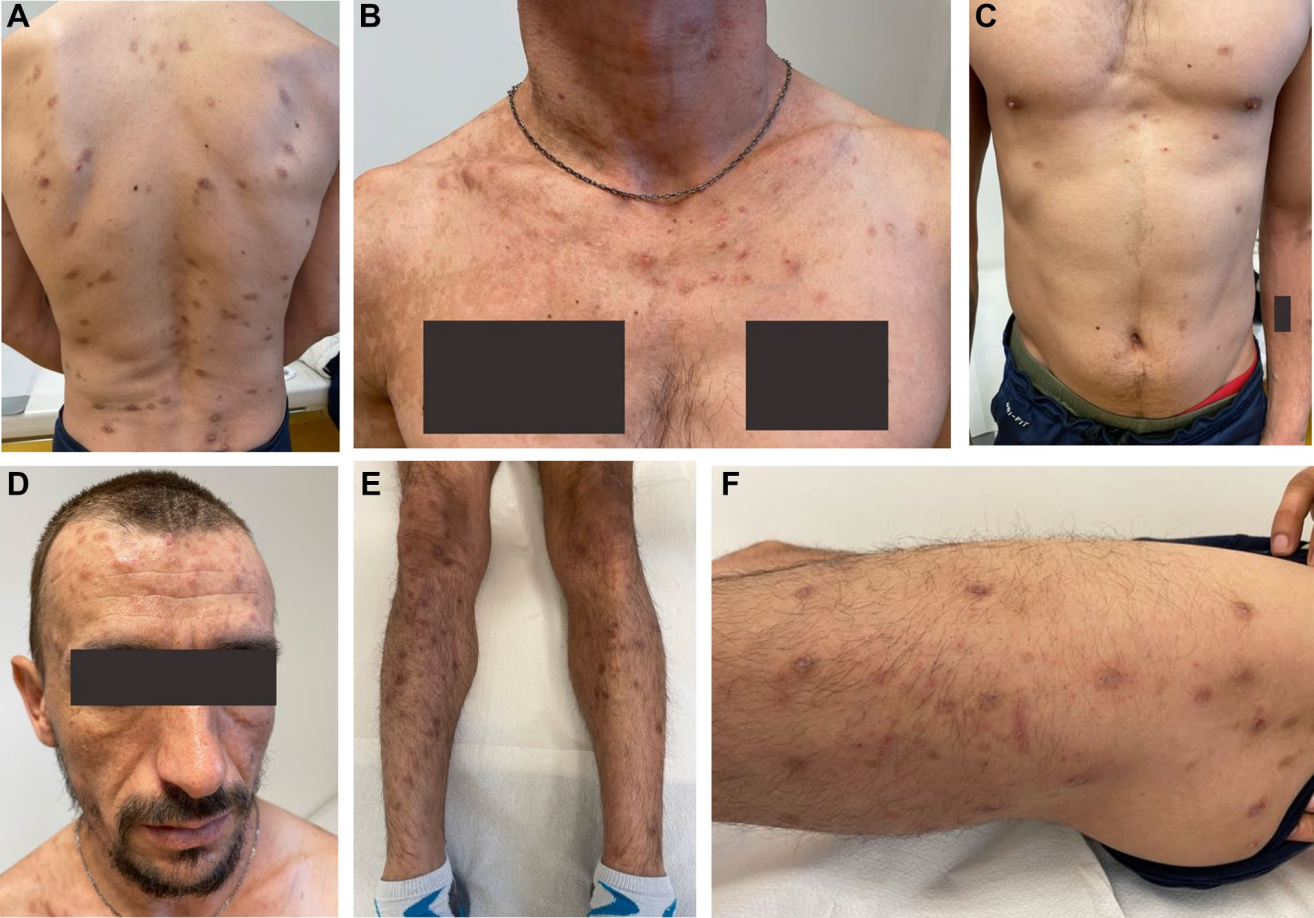
**A**–**F** Clinical course after second course of treatment with Valaciclovir after explanation of medication compliance by a native speaker

## Data Availability

Not applicable.
